# Two-Stage Wildlife Event Classification for Edge Deployment

**DOI:** 10.3390/s26041366

**Published:** 2026-02-21

**Authors:** Aditya S. Viswanathan, Adis Bock, Zoe Bent, Mark A. Peyton, Daniel M. Tartakovsky, Javier E. Santos

**Affiliations:** 1 Department of Energy Science and Engineering, Stanford University, Stanford, CA 94305, USA; tartakovsky@stanford.edu; 2 Pajarito Environmental Education Center, Los Alamos, NM 87544, USA; adisbock@gmail.com; 3 Rubenstein School for Environment and Natural Resources, University of Vermont, Burlington, VT 05405, USA; zoe.bent@uvm.edu; 4 Department of Fish, Wildlife and Conservation Ecology, New Mexico State University, Las Cruces, NM 88003, USA; nmcatmap@nmsu.edu; 5 Los Alamos National Laboratory, Earth and Environmental Sciences Division, Los Alamos, NM 87545, USA; jesantos@lanl.gov

**Keywords:** edge computing, real-time environmental monitoring, computer vision, artificial intelligence, object detection, image classification, curriculum learning

## Abstract

Camera-based wildlife monitoring is often overwhelmed by non-target triggers and slowed by manual review or cloud-dependent inference, which can prevent timely intervention for high stakes human–wildlife conflicts. Our key contribution is a deployable, fully offline edge *vision sensor* that achieves near-real-time, highly accurate wildlife event classification by combining detector-based empty-image suppression with a lightweight classifier trained with a staged transfer-learning curriculum. Specifically, Stage 1 uses a pretrained You Only Look Once (YOLO)-family detector for permissive animal localization and empty-trigger suppression, and Stage 2 uses a lightweight EfficientNet-based binary classifier to confirm puma on detector crops and gate downstream actions. Our design is robust to low-quality nighttime monochrome imagery (motion blur, low contrast, illumination artifacts, and partial-body captures) and operates using commercially available components in connectivity-limited settings. In field deployments running since May 2025, end-to-end latency from camera trigger to action command is approximately 4 s. Ablation studies using a dataset of labeled wildlife images (pumas, not pumas) show that the two-stage approach substantially reduces false alarms in identifying pumas relative to a full-image classifier while maintaining high recall. On the held-out test set (N=1434 events), the proposed two-stage cascade achieves precision 0.983, recall 0.975, F1 0.979, accuracy 0.986, and balanced accuracy 0.983, with only 8 false positives and 12 false negatives. The system can be easily adapted for other species, as demonstrated by rapid retraining of the second stage to classify ringtails. Downstream responses (e.g., notifications and optional audio/light outputs) provide flexible actuation capabilities that can be configured to support intervention.

## 1. Introduction

Camera traps and other camera-based sensing systems are now widely used for environmental monitoring, biodiversity assessment, and wildlife management [1,2]. Tools such as Megadetector [3] and Wildlife Insights [4] are designed to help scientists analyze trail camera images for research and can perform complex classification tasks that can differentiate between multiple species. These efforts are typically done weekly or monthly in batch mode and do not require rapid inference [5,6]. While such latency is acceptable in many ecological studies, there are a growing set of operational contexts where the decision window can be minutes rather than days.

Human activities are reshaping natural ecosystems, resulting in habitat loss and imposing significant pressures on large carnivores [7]. In addition, large scale ecological disturbances such as droughts and wildfires driven by climate change are transforming landscapes and altering wildlife distributions, further exacerbating human–wildlife interactions [8,9]. In the case of large mammals entering human-used spaces (e.g., polar bears in towns), wildlife approaching transportation corridors or other infrastructure (e.g., elephants near railway lines), and rare or high-stakes events in insufficiently protected areas (e.g., pumas attacking livestock), near-real-time detection, of the order of a few seconds, is of the utmost importance in order to enact intervening measures [10].

Field deployment requiring real-time or near real-time response faces several challenges, the most important consideration being a fast yet accurate classification algorithm. Additionally, deployments often operate in remote locations with intermittent connectivity, constrained budgets, minimal power and limited compute [11]. This requires edge computing approaches that run inference locally rather than relying on cloud processing. Furthermore, the data itself presents difficulties: large fractions of motion-triggered captures may be empty, and animal-containing images can be low resolution and hard to interpret automatically, especially in low light situations [12,13]. Nighttime trail camera imagery is often monochrome/infrared (IR) and frequently affected by motion blur, illumination artifacts, and partial-body captures; in contrast, daytime color images typically contain richer information and sharper images. Practical systems must therefore work across both night and day conditions while suppressing empty triggers to avoid excessive false alarms. Labeled data can also be limited in deployment-specific settings, motivating approaches that reduce relabeling burden while maintaining reliability [14].

There is no single state of the art vision sensing AI model that performs efficiently and reliably under all of the above-mentioned constraints. While frontier multimodal models are highly capable, they are typically accessed as externally hosted, proprietary services, which introduces ongoing cost, connectivity requirements, and variable end-to-end latency, complicating guarantees of seconds-scale response in remote deployments [15,16,17,18]. Even when running locally, a model often has to do two different jobs at once: (i) ignore the many empty and nuisance triggers from wind, rain, etc., and (ii) make a high-confidence, target-species decision when an animal is present. Edge-friendly tools such as YOLO [19,20] are excellent at quickly finding animal-like image regions but have poor reliability in confirming a specific target species. This is exacerbated when the animal may be distant, partially visible, and frequently captured in infrared at night. These gaps are especially important in near-real-time workflows where repeated false alarms quickly undermine trust and usability.

Beyond the YOLO family, other lightweight single-shot detector families such as SSD [21] and EfficientDet [22] are widely used as embedded-friendly options for object detection under constrained compute budgets. In our low-contrast night-IR setting, we found that such single-shot detectors can be brittle when the target animal occupies only a small fraction of the image, as contrast loss and background clutter make localization unstable. This motivates our two-stage design that first detects candidate wildlife events and then applies a more selective classification step. In principle, neural-operator approaches (e.g., FNO-style models [23,24]) could further reduce the classifier’s sensitivity to image resolution while remaining computationally efficient. However, in our experiments EfficientNet already provided a satisfactory accuracy for binary classification even if the cropped region was small and pixelated. Separately, a common strategy in wildlife monitoring is to exploit temporal context (bursts or video) using CNN → RNN pipelines (e.g., LSTM/GRU variants) to stabilize predictions across images [25,26]. While temporal aggregation can improve robustness when reliable sequences are available, they are more time consuming than inference on single images. Our deployment requires rapid on-site decisions within a short interaction window (often only a few seconds, e.g., ∼4 s) with severe night-IR degradations. Buffering multiple images for temporal modeling increases end-to-end latency and system complexity on embedded hardware which we wanted to avoid. We therefore focus on an event-level, two-stage still-image cascade that is robust without assuming temporal continuity.

To address these gaps, we present a detect–classify cascade, a two-stage AI-enabled *vision sensor* that forms the core of our near-real-time environmental monitoring system. The system itself consists of an event-driven camera paired with an edge-computing unit that performs on-device inference and triggers user-defined responses. Related smart camera-trap systems demonstrate on-device inference and integrated field prototypes [27,28]; here, we focus on seconds-scale, species-specific identification for high-stakes events. We demonstrate our approach for a sample dataset containing camera trap images, distinguishing pumas from other wildlife. We report end-to-end trigger-to-action latency and false-trigger behavior across both daytime color and nighttime infrared imagery. Finally, we include Grad-CAM visualizations [29] for explainability of classifier decisions that build trust in the system and guide model refinement for terrain and species specific applications.

Our primary innovation is a two-stage pipeline: a permissive first-stage detector paired with a second-stage classifier trained via a curriculum-learning approach. This design fills a niche required to meet our constraints of challenging nighttime infrared conditions and seconds-scale inference on edge hardware. The result is reliable, on-device identification while the animal is still on-site, enabling real-time notifications and optional audio/light deterrents. We also incorporate classifier explainability to improve robustness. By inspecting which features drive decisions and where classification fails, we can systematically identify failure modes and feed those examples back into our data collection and training pipeline, improving performance over time.

Our contributions can be summarized as follows:

- A novel two-stage edge-deployable pipeline that uses a Stage 1 detector and a staged transfer-learning curriculum for Stage 2 species confirmation;
- An interpretability workflow using Grad-CAM visualizations to surface failure modes and edge cases (e.g., false triggers, partial-body/night-IR errors) and guide iterative model refinement for field deployments;
- A deployable, offline *vision sensor* that integrates motion-triggered acquisition, on-device inference, and user-defined actions on low-cost edge hardware designed for challenging field imagery;
- An openly released, re-trainable implementation (code, weights, labeled datasets, and a hardware bill of materials) designed for field use.

The system is designed with flexibility in mind, to be extended to other species with modest additional labeled data. While our quantitative evaluation emphasizes pumas as a high-stakes management case study, we also include an illustrative ringtail deployment obtained by retraining only Stage 2 with a few hundred labeled images, demonstrating that the same pipeline can be adapted to new target species with modest additional data.

The remainder of the paper is organized as follows: Section 2 describes the system architecture and two-stage methodology; Section 3 details the case study and evaluation protocol; Section 4 presents quantitative results and ablations; Section 5 discusses implications, limitations, and failure modes; and Section 6 summarizes the key conclusions.

## 2. Materials and Methods

### 2.1. System Overview

**Figure 1:**
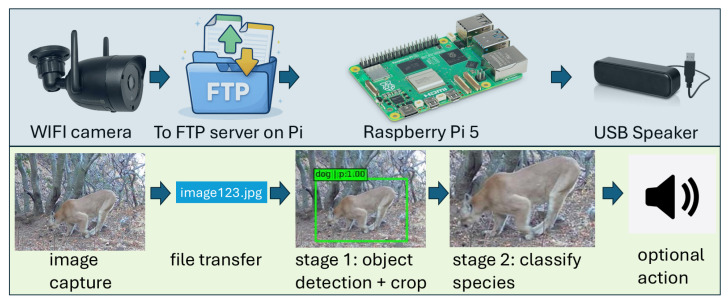
AI-enabled vision sensor workflow for near-real-time environmental monitoring: (i) An event triggers image acquisition at the sensor; (ii) images are transmitted over a local network to an edge device; (iii) the edge device performs on-device inference to detect/classify objects of interest; and (iv) when predefined criteria are met, a downstream action (e.g., alert, logging, or actuator command) is initiated. Row 1 shows the physical hardware components and Row 2 shows the associated data flow and processing steps/actions.

We introduce an AI-enabled *vision sensor* for near-real-time wildlife monitoring that couples event-driven image acquisition with two-stage edge inference and configurable downstream actions. The system operates in an event-triggered manner: a motion trigger activates image capture, and the resulting image(s) are transmitted over a local network to an edge-computing device for seconds-scale inference and decision-making. Figure 1 summarizes the end-to-end pipeline, spanning (i) event triggering and image acquisition, (ii) data transfer, (iii) two-stage edge inference, and (iv) action generation. The system is designed to operate without internet connectivity (offline inference), and run on low-cost, low-power hardware suitable for field deployment without access to mains power. The design goal is for the system to achieve seconds-scale end-to-end latency to support real-time intervention [10].

### 2.2. Two-Stage Classification

**Figure 2:**
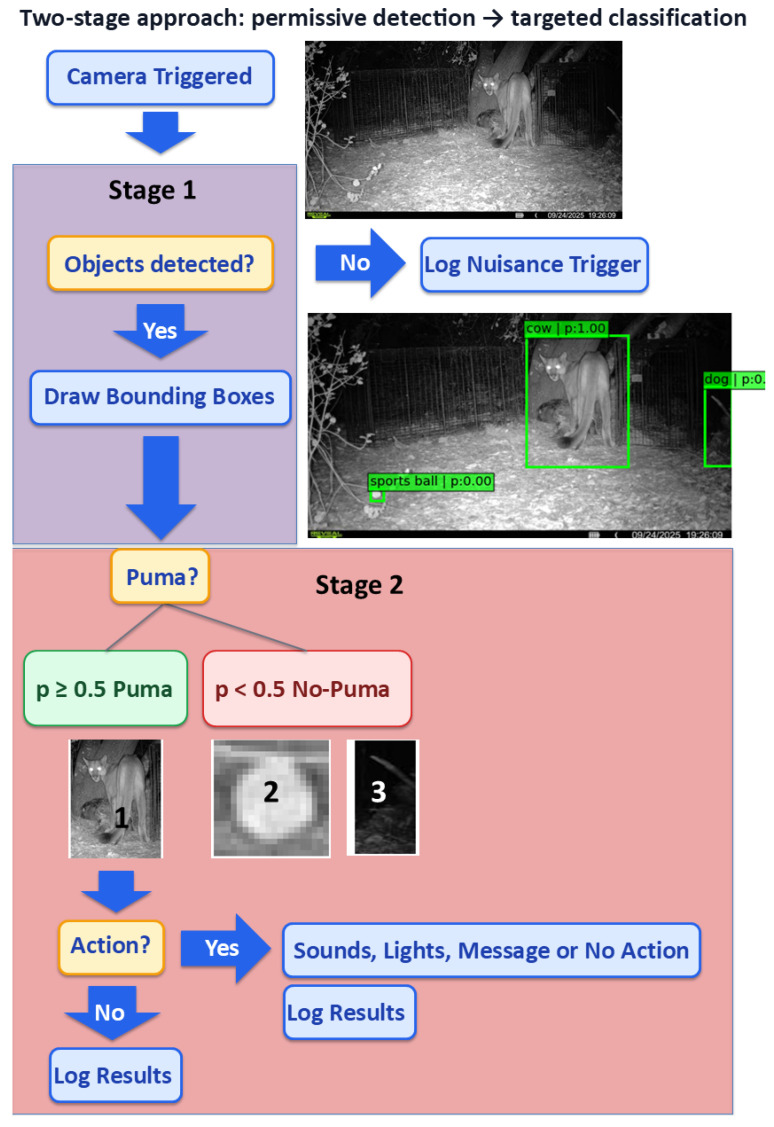
Two-stage detect–classify cascade. In Stage 1, a high-recall permissive detector rejects obvious non-events and empty images while identifying an object region of interest (ROI) for cropping. In Stage 2, a computationally efficient binary classifier evaluates the ROI to verify if puma and triggers optional downstream actions.

Our central methodological contribution is a two-stage detector–classifier cascade designed for reliable, species-specific decisions under resource constraints (Figure 2) described below in detail.

#### 2.2.1. Stage 1: YOLO for Real-Time Localization and Trigger Suppression

We use a one-stage detector (YOLO) [19] in Stage 1 to meet on-device latency and compute constraints while localizing candidate animals. Specifically, we use YOLOv8, a well-supported real-time object detector with an accessible training and deployment workflow [30]. In our setting, Stage 1 is used as a high-recall localizer and empty/nuisance-image suppressor: it filters motion-triggered images with no relevant content and produces animal detections that are cropped into Regions of Interest (ROIs) for downstream classification. Because detector class labels can be unreliable under trail-camera conditions (e.g., nighttime IR, motion blur, and partial-body captures), we avoid treating Stage 1 outputs as the final species decision and instead use a dedicated confirmation stage.

#### 2.2.2. Stage 2: EfficientNet with Curriculum Learning for ROI Classification

Stage 2 confirms the target species from the ROIs and gates downstream actions. We implement Stage 2 with an EfficientNet classifier [31], using EfficientNetV2-S for binary (puma vs. no-puma) classification due to its favorable accuracy–efficiency trade-offs. Training follows a two-step curriculum. In Step 1, we initialize EfficientNetV2-S with ImageNet-pretrained weights [32], replace the ImageNet classification head with a binary head, and train only the new head while freezing the backbone to transfer generic visual features to our domain. In Step 2, we fine-tune all layers on a balanced, species-specific dataset spanning diverse lighting, seasons, and camera viewpoints. This staged optimization improves convergence stability and reduces overfitting and catastrophic forgetting, yielding higher accuracy than single-phase training.

This two-stage cascade follows the principle that sequential pipelines that filter and refine candidates, relative to single-shot approaches, can improve robustness [33]. This structure also supports reuse: Stage 1 functions as a general animal-localization module, while Stage 2 can be retrained for a new target species with modest labeled data. A detailed ablation study in Section 4 demonstrates the need for this two-stage cascade.

### 2.3. Hardware Platform

*Camera and triggering*: Images are acquired using a File Transfer Protocol (FTP)-enabled Wireless Fidelity (WiFi) security camera with a Passive InfraRed Sensor (PIR) for motion triggering. In our implementation we used a MICROSEVEN M7B1080P-WSAA (Microseven Systems LLC, Walnut, CA, USA), but the approach is designed to be compatible with many commercially available FTP-capable security cameras (e.g., several Reolink models) that can upload triggered images to a local FTP server. These cameras typically provide configurable motion/PIR detection zones, which helps reduce nuisance triggers from irrelevant motion (e.g., wind-driven vegetation or traffic outside the area of interest) and improves the practicality of event-driven monitoring in the field.

*Edge-computing device and networking*: A Raspberry Pi 5 serves as the edge-computing device and runs the on-device inference pipeline and action policy. The system supports two networking configurations: (i) a standalone mode where the Raspberry Pi 5 hosts a local WiFi hotspot that the camera(s) join, and (ii) a site-network mode where the Raspberry Pi 5 and camera(s) join an existing WiFi network (common at the urban–wildland interface). In both modes, cameras upload triggered images to the Raspberry Pi 5 via FTP, and the inference workflow is identical. The system does not require external internet access once models are trained.

*User interface*: Users interact with the system by connecting a laptop or phone to the same WiFi network that the camera(s) are on. The connection is established through a platform-independent graphical user interface implemented using the Flutter framework [34]. The user interface supports monitoring from devices in either of the two supported network modes. Users connect directly to the Raspberry Pi 5 to view images, download them for offline use, delete images that are not needed, and adjust system settings such as detection parameters and optional audio-file selection. Users can adjust a small number of settings in the interface, including detector sensitivity, and whether audio is enabled.

*Outputs (optional)*: Downstream outputs are configurable and can include (i) notifications for rapid stakeholder awareness and (ii) optional on-site cues such as lights and/or audio to support immediate event verification. These output modalities are widely discussed in the deterrence literature [35,36], but here they are treated as actuation capabilities rather than validated interventions.

*Power considerations*: In a single-camera configuration with a USB speaker, average total power draw is approximately 8 W (Table 1). In many intended use cases (e.g., homes, barns, ranches), power is readily available. For deployments in remote/wild areas, the system can be powered using a battery generator and/or solar panel system sized for the expected duty cycle and local conditions. The camera contributes substantially to the overall power budget because it captures continuous video and, at night, powers infrared illumination. This feature is currently retained as it is beneficial for our current research and monitoring needs. However, reducing camera power draw is a practical direction for future optimization; for example, camera models or configurations that allow enabling video only during certain times (while retaining event-driven image capture/upload) could reduce overall system power consumption.

*Multi-camera deployments*: Our approach supports multiple FTP-enabled WiFi cameras connecting to the same Raspberry Pi 5 single-board computer (Raspberry Pi Ltd, Cambridge, UK) hotspot, allowing coverage to be expanded by adding cameras. We tested the system with up to three cameras connected concurrently. If multiple cameras upload images simultaneously, incoming images are placed into a queue and processed sequentially by the inference pipeline. Because on-device inference is fast (sub-second per image for each stage) and target events are typically sparse in time for many monitoring scenarios, queuing is expected to have limited impact on overall system responsiveness in typical deployments; however, sustained high trigger rates could increase latency due to backlog. Power demand scales approximately with the number of cameras (and any speaker usage), and can be estimated as in Equation (1)

(1) $$P_{\mathrm{total}}\approx P_{\mathrm{Pi}}+N_{\mathrm{cam}}\;P_{\mathrm{cam}}+P_{\mathrm{spk}}.$$

Here, $P_{\mathrm{total}}$ is total system power, $P_{\mathrm{Pi}}$ is the Raspberry Pi power draw, $N_{\mathrm{cam}}$ is the number of cameras, $P_{\mathrm{cam}}$ is per-camera power draw, and $P_{\mathrm{spk}}$ is the (optional) speaker power draw.

*Enclosure and cost*: The prototype uses commercially available components to minimize build complexity and cost. In our current configuration, the Raspberry Pi 5 costs approximately $60, the FTP-enabled WiFi (PIR) camera costs approximately $40, and the USB speaker costs approximately $15. The camera and speaker are weather-resistant/waterproof for outdoor deployment, while the Raspberry Pi 5 is housed in a waterproof enclosure (approximately $15). Thus, the total hardware cost for a single-camera configuration with optional audio output is approximately $130 (excluding power supply/battery/solar components).

### 2.4. Software Pipeline

*Data flow and orchestration*: As described in Figure 1, the system operates in an event-triggered manner. Motion-triggered images are uploaded via FTP and written to a directory on the Raspberry Pi 5 which is monitored by a lightweight file-watcher utility. New arrivals are detected and queued for processing in a sequential manner when multiple cameras upload concurrently. This ensures that peak resource use remains bounded. For each processed image (and each downstream ROI), the system records structured metadata—including timestamp, camera identifier, detector outputs, classifier scores, final decision, and any action taken—supporting later analysis and auditing. To improve robustness in field deployments, corrupted or partially uploaded files are detected and skipped (logged and ignored) rather than propagating errors that could halt the pipeline.

#### 2.4.1. Stage 1: Object Detection (Empty-Image Suppression and ROI Cropping)

We use an Ultralytics YOLOv8s detector with pretrained weights (yolov8s.pt) running on the Raspberry Pi 5. Inference uses image size = 640 with a YOLO confidence threshold of 0.25 and YOLO non-maximum suppression (NMS) IoU (intersection-over-union) threshold of 0.45. If no detections exceed the confidence threshold, the image is treated as an empty/non-target trigger and is not forwarded to Stage 2. For each retained detection, we generate an ROI by expanding the bounding box by 15% padding (CROP_EXPAND = 0.15) prior to cropping. All detected boxes are forwarded to Stage 2 without detector-class filtering; this choice reduces the risk of suppressing true events when detector labels are unstable under challenging infrared or partial-body conditions.

#### 2.4.2. Stage 2: Curriculum Learning for Binary Classification

Each Stage 1 ROI is classified using an EfficientNetV2-S binary classifier initialized with ImageNet weights. Inputs are resized to (384, 384, 3) and processed using the model’s built-in EfficientNetV2 preprocessing (include_preprocessing = true); no additional infrared-specific preprocessing is applied beyond this shared pipeline. At the time of deployment, the classifier outputs a confidence score, which is thresholded to produce a true event vs. not-true event decision. Implementation details and full training/configuration hyperparameters are provided in the accompanying repository (see the Data Availability statement).

The system decides what to do after each camera trigger using a simple set of rules. Stage 1 may produce zero or more ROIs from a triggered image. Each ROI is scored by Stage 2, which outputs a true event probability. We classify an event as true event if any ROI has a probability ≥0.5; otherwise, the event is not-true event. All results in this work are based on a threshold of 0.5.

## 3. Case Study

### 3.1. Study Context and Dataset

The species chosen for this demonstration was the puma. Pumas are wide-ranging large carnivores with important ecological roles [37,38] and are also implicated in management challenges near the wildland–urban interface and around domestic animals. Management actions such as lethal removal can have complex outcomes, motivating scalable nonlethal approaches and improved monitoring tools [39,40]. Selective sensing can enable targeted downstream actions, including activation of well-established deterrent modalities, once reliable low-false-alarm triggering is available [35,36]. The scope of this study is limited to the sensing-and-classification capability only.

*Study region*: The original training and validation dataset was assembled by the Large Mammal Monitoring Project [2,41]. The project investigates the effect of climate change, drought, and wildfire on large mammal numbers and behavior by collecting trail camera imagery in the Pajarito Plateau area of New Mexico. Because most labeled training and validation images come from this single region, there is potential geographic concentration bias (e.g., habitat- or site-specific background cues) that could reduce performance when deployed in substantially different environments. Dozens of trail cameras have been deployed, capturing thousands of images of pumas and other wildlife. The region includes rugged canyons and mesas and supports diverse wildlife, including deer, elk, bears, bobcats, coyotes, and pumas. The resulting imagery contains animals approaching from multiple angles and distances under varying lighting conditions. Additionally, data were collected using multiple trail camera brands with different imaging sensors and brightness profiles, improving robustness to cross-camera domain shifts. We further augmented this dataset with images collected from our own field deployments in New Mexico and California to better handle edge cases.

*Imaging conditions*: Our dataset spans both nighttime infrared imagery and daytime color imagery. Nighttime IR images are often lower quality and more prone to motion blur and illumination artifacts, whereas daytime color images typically contain richer information and sharper images. Including both modalities supports around-the-clock monitoring and improves practical deployability. Training, validation and test datasets each contain images across the entire spectrum of operating conditions.

*Dataset and Labels*: For training and validation, we draw from a dataset that consists of 1103 puma images and 1693  no-puma images using an 80/20 split. For test data we use an independent set of 479 puma and 955 no-puma images. Importantly, this test set was collected from deployments in California and is geographically disjoint from the New Mexico training/validation data; it includes different background vegetation/terrain and includes camera hardware not fully overlapping with the training set, providing a direct cross-site (and partially cross-camera) domain-shift evaluation of generalization for pumas. Because camera-trap deployments are dominated by non-target triggers, the resulting dataset is moderately imbalanced toward the no-puma class. We therefore use a class-stratified 80/20 split for training/validation and verify that both daytime and nighttime conditions are represented across train/validation/test. This imbalance motivates reporting balanced accuracy in addition to accuracy and error rates. We manually verified that the range of imaging conditions is captured in the sets. We formulated a binary classification task: puma vs. no-puma. The no-puma class includes other wildlife species (e.g., foxes, coyotes, bobcats, bears, deer, elk, skunk) as well as empty or nuisance-trigger images (e.g., wind-driven vegetation, precipitation, insects, and other non-animal motion). Labels were assigned at the image level based on visual review: an image was labeled puma if any part of a puma was visibly present (including partial-body views), and labeled no-puma otherwise. This binary labeling scheme is also straightforward to adapt to other target species, because it only requires image-level labels for a single target class versus a pooled no-target class rather than exhaustive multi-species annotation.

### 3.2. Prototype Evaluation

We evaluate the system for near-real-time monitoring in terms of its speed and reliability. We first conduct an offline ablation study of the two-stage sensing-and-classification methodology developed here. This is performed by systematically testing individual parts of the cascade to compare the performance by dividing the dataset into the same training and test categories for each step. We also validate the entire pipeline in field deployment mode to ensure that not only does the two-stage classification work accurately within seconds but that the entire end-to-end pipeline functions as intended.

*Baselines and ablations*: We use an independent test dataset previously unseen by the classifier, with 479 puma images and 955 no-puma images to perform our ablation study. This held-out set is geographically disjoint from training/validation (California vs. New Mexico) and therefore serves as our primary quantitative check on cross-site generalization for puma detection. We perform the following ablations:

- Stage 1 only (YOLO label proxy)—detector-only baseline that predicts puma if the pretrained YOLO detector reports a cat detection; otherwise no-puma.
- Stage 2 only (full image)—single-stage baseline that applies the curriculum-learned binary EfficientNet classifier directly to the uncropped original image.
- Two-stage (animal-only filter)—ablation in which Stage 1 detections are filtered to a restricted “animal” label set before cropping; Stage 2 classifies the resulting ROIs.
- Two-stage (proposed; permissive Stage 1)—the proposed detector → classifier cascade, where Stage 1 is used permissively for localization/cropping and empty-trigger suppression, and Stage 2 performs puma confirmation on the ROIs and gates downstream actions.

### 3.3. Metrics

The following questions guide our evaluation of the system:

1. How accurately does the pipeline distinguish target vs. non-target across nighttime infrared and daytime color conditions?
2. How effectively does the two-stage cascade suppress false triggers (empty images and non-target wildlife) compared to simpler baselines?
3. What is the end-to-end latency from trigger to action command, and how does it decompose by stage?

We record True Positive (TP), True Negative (TN), False Positive (FP) and False Negative (FN) classifications. Because raw FP/FN counts depend on dataset size and class balance, we report them for operational context and pair them with normalized rates and standard metrics. Offline performance is quantified using precision (P), recall (R), F1 score (F1), accuracy (Acc), balanced accuracy (BalAcc), false positive rate (FPR), and false negative rate (FNR), where

(2) $$P=\frac{TP}{TP+FP},\;R=\frac{TP}{TP+FN},\;F1=\frac{2PR}{P+R}.$$

**Figure 3:**
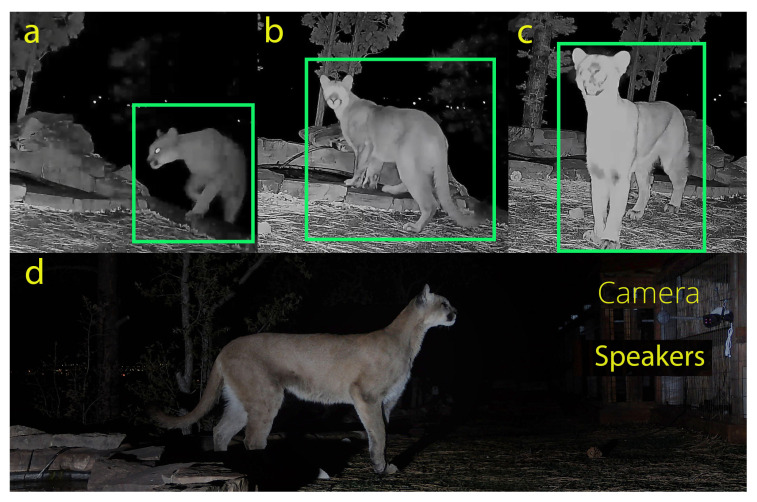
Field deployment example illustrating the end-to-end sensing-to-action workflow. A puma approaches a residential pond (19 May 2025): (**a**) motion-triggered images are acquired and the animal is localized (green bounding boxes) and classified as puma in near real time (20/21 images classified correctly in this encounter); (**b**) an audio output is triggered after classification to demonstrate actuation capability; (**c**) the puma orients toward the sound source; (**d**) DSLR photo of the puma during the event. This figure is intended as a qualitative demonstration of integrated operation (trigger → transfer → inference → action), not as a controlled evaluation of behavioral deterrence. The full video is available at https://vimeo.com/1087210081 (accessed on 17 February 2026).

We additionally compute

(3) $$\begin{array}{cc} Acc & =\frac{TP+TN}{TP+TN+FP+FN},\\ BalAcc & =\frac{1}{2}\left(\frac{TP}{TP+FN}+\frac{TN}{TN+FP}\right),\\ FPR & =\frac{FP}{FP+TN},\\ FNR & =\frac{FN}{FN+TP}. \end{array}$$

for the positive class (puma) in the confusion matrices reported in Equations (4)–(7). Operational performance is characterized by (i) end-to-end latency and its stage breakdown (Section 4.4) and (ii) qualitative field demonstrations of the integrated workflow (trigger → transfer → inference → action; Figure 3).

End-to-end latency is measured as the elapsed time from camera trigger (image timestamp at upload) to issuance of an action command by the edge device (e.g., notification event and/or initiation of audio playback). Latency values were computed over 100 triggered events collected during field operation across both networking configurations.

## 4. Results

### 4.1. Offline Performance Evaluation (Ablation and Baselines)

We begin with an offline ablation study to isolate the role of each stage in the two-stage pipeline. The goal is straightforward: to show what breaks if we skip parts of the system. These comparisons motivate the full two-stage cascade and explain why seemingly sensible shortcuts can lead to more missed events or more false alarms, both of which lead to different undesirable consequences and lowered trust.

#### 4.1.1. Stage 1 Only (YOLO Label Proxy)

We evaluate a detector-only baseline using YOLO, a common choice for fast edge deployment. Because puma is not a dedicated detector class in our YOLO model, we use a proxy rule by flagging puma whenever YOLO reports cat (Equation (4)). Under this setting, the model correctly identifies 160/479 puma events and correctly rejects 948/955 no-puma events. This corresponds to a precision of 0.958, recall of 0.334, and F1 of 0.495 (puma treated as the positive class). Furthermore, even when the detector localizes the animal, its predicted class label is unreliable under field conditions: motion-triggered wildlife imagery (often infrared, blurred, and partially occluded) is frequently assigned to visually adjacent or even unrelated categories. The Confusion matrix for the Stage 1 only (YOLO label proxy) baseline is:

(4) $$C_{stage1}=\left[\begin{array}{cc} 948 & 7\\ 319 & 160 \end{array}\right]$$

#### 4.1.2. Stage 2 Only (Full-Image Curriculum-Learned Classifier)

We also evaluate a classifier-only baseline (EfficientNet on the full image) and show that, without detector-based localization and empty-image filtering, it produces more false alarms in cluttered, motion-triggered imagery. Equation (5) shows that applying the classifier directly to full images yields relatively high sensitivity to pumas (FN = 33), but at the cost of many false positives (FP = 171). Equation (5) makes this tradeoff explicit: the model correctly identifies 446/479 puma events (recall = 0.931), but incorrectly flags 171/955 no-puma events as puma (precision = 0.723). The Confusion matrix for the Stage 2 only (full image) baseline is:

(5) $$C_{stage2}=\left[\begin{array}{cc} 784 & 171\\ 33 & 446 \end{array}\right]$$

#### 4.1.3. Two-Stage with an Animal-Only Filter

A natural idea is to reduce clutter by filtering Stage 1 detections to “animal” labels before passing ROIs to Stage 2. Equation (6) shows that this strategy produces substantially more missed pumas than full-image classification (FN = 89 vs. 33). Only 390/479 puma events are correctly identified (recall = 0.814), while 89 puma events are filtered or rejected as no-puma before Stage 2 can identify them. The reason is that the detector’s semantic labels are less reliable than its ability to localize an object: many true pumas are localized when all classes are enabled but are assigned non-animal (or otherwise incorrect) labels and therefore get removed by the label filter. Once those images are excluded, Stage 2 never has the opportunity to correct the detector’s label error. The Confusion matrix for the two-stage (animal-only filter) ablation is:

(6) $$C_{animal_{f}ilter}=\left[\begin{array}{cc} 948 & 7\\ 89 & 390 \end{array}\right]$$

#### 4.1.4. Proposed Two-Stage Workflow

Equation (7) shows the proposed design, keeping Stage 1 permissive for localization and empty-trigger suppression, then using Stage 2 for the puma decision on cropped ROIs. This configuration achieves both low false alarms (FP = 8) and low missed events (FN = 12), improving substantially over the individual stages. The confusion matrix in Equation (7) summarizes this balance: 467/479 puma events are correctly detected while only 8/955 no-puma events are incorrectly flagged. The reduction in false alarms is driven by Stage 1 suppressing empty or nuisance triggers and restricting Stage 2 to animal-containing ROIs, which removes much of the background clutter that confuses a full-image classifier. The few remaining misses are dominated by cases where Stage 1 fails to localize the animal (e.g., low contrast with foliage in nighttime infrared imagery), highlighting that localization quality is the primary limiting factor once the two-stage cascade is used. The Confusion matrix for the proposed two-stage workflow is:

(7) $$C_{2stage}=\left[\begin{array}{cc} 947 & 8\\ 12 & 467 \end{array}\right]$$

### 4.2. Sensitivity to Stage 1 and Stage 2 Model Choice

We next evaluate whether substituting newer model families in Stage 1 (object detection) and Stage 2 (binary classification) changes offline performance, or whether the primary driver is the two-stage pipeline itself.

#### 4.2.1. Stage 1 Only (YOLO Label Proxy): YOLOv8 vs. YOLO26

We compare our current Stage 1-only baseline (YOLOv8 [30] label proxy; Equation (4)) against the newer YOLO26 [42] under the same proxy rule (flag puma whenever the detector reports cat). Table 2 summarizes results. Performance is similar, consistent with the interpretation that Stage 1 is primarily used to cast a wide net and provide candidate ROIs for Stage 2. Latency varies slightly across images (with the number of predicted bounding boxes), so we report average latency; under this measure, YOLO26 is slightly faster than YOLOv8.

#### 4.2.2. Stage 2 Only (Full-Image Classifier): EfficientNet vs. ConvNeXt-Tiny

We also compare the Stage 2-only baseline reported in Equation (5) (EfficientNet [31] applied to the full image) against a newer ConvNet backbone (ConvNeXt-Tiny [43]) trained with the same curriculum and evaluation protocol. Table 3 shows similar full-image performance. This supports the interpretation that Stage 2 is most effective when applied to ROIs provided by Stage 1, where it can suppress false positives that arise in cluttered full-image imagery. We also report average inference latency (in seconds) on our Raspberry Pi deployment. Unlike Stage 1 detection, Stage 2 latency is fairly consistent across crops because each ROI is resized to a fixed input resolution. EfficientNet is slightly faster in our setup, consistent with its lower computational footprint, while ConvNeXt-Tiny was not specifically designed for edge deployment.

Across both stages, substituting newer architectures yields similar offline performance under the same evaluation protocol. The limited sensitivity to architecture choice is consistent with our motivation for using the two stage approach: once Stage 1 reliably proposes candidate ROIs, the remaining errors are driven more by challenging imagery (motion blur, low contrast, partial views) and the ROI-vs-full-image distinction than by the specific detector or backbone family. Because both YOLOv8/YOLO26 and EfficientNet/ConvNeXt are strong pretrained models, differences are expected to be small when the training data and protocol are held constant.

In addition, for our hardware, the latency of the new models is also similar to the older models. Although YOLO26 is slightly faster than YOLOv8 in our measurements by ≈0.1 s, our current end-to-end bottleneck is not model inference. The dominant contributor to total turnaround is transferring each image from the camera to the Raspberry Pi via FTP, which accounts for most of the ∼4 s cycle time. Consequently, further reducing model latency would have limited impact on end-to-end responsiveness. Given the small differences and the importance of reproducibility in long-running deployments, we retain our original YOLOv8 and EfficientNet choices for the remainder of this study, as they are well-tested and stable within our training and inference pipeline.

### 4.3. Field Deployment and Operational Workflow Validation

We have continuously deployed the system since May 2025 in the field to validate end-to-end robustness under realistic triggering conditions (wind, precipitation, insects, and non-target wildlife) and to confirm that the sensing-to-action loop runs without external internet access. Deployments were conducted at two sites using both supported networking configurations, using an existing WiFi network, and in standalone mode, with a hotspot. In both modes, cameras upload motion-triggered images via FTP to the Raspberry Pi and downstream inference and action logic is identical. In the field, the trigger stream is dominated by empty or weather-disturbed images, with non-target animals (e.g., skunks, ringtails, raccoons, and foxes) far more common than puma visits. On nights with calm weather, the system ingests on the order of ∼100 motion-triggered events, whereas windy, rainy, or snowy conditions can generate ∼1000 events due to nuisance motion. These conditions provide a realistic stress test of false-trigger suppression and alert gating under heavy background activity. Across ongoing deployments comprising tens of thousands of motion-triggered events, we quantified to be 0.8% from 98 false puma detections out of 12,436 triggers, where a false positive is defined as a non-puma event that nonetheless produces a puma alert/action. False puma detections were a combination of animals such as foxes posed in ways that resemble a puma. Other false detections result from ROIs of inanimate objects that resembled a puma.

Because puma events are rare at our sites, these deployments are primarily informative for operational robustness and false-trigger behavior. Field operation discovered systematic edge cases (e.g., animals that resemble pumas at certain angles) that are underrepresented in curated offline splits. We used these field observations to refine labeling guidelines for ambiguous triggers and to prioritize targeted data collection (hard negatives and rare positive contexts), strengthening the training/validation coverage and the representativeness of held-out evaluation data used for the offline results reported in Section 4.1. Across continuous operation, we observed a small number of site-network infrastructure interruptions (two power outages and four temporary WiFi outages), all of which resolved without manual intervention as the Raspberry Pi rebooted and services resumed automatically, providing practical evidence of robustness to common field failures.

#### 4.3.1. End-to-End Workflow Demonstrations

We validated the integrated workflow (trigger → transfer → inference → action) using qualitative field demonstrations in which an audio output was triggered following target detection. Figure 3 shows an example puma encounter from the site-network deployment (19 May 2025) where a caterwauling call was played to get a response from the puma without making it a nuisance or scaring it away. This encounter demonstrates Stage 1 localization (bounding boxes), Stage 2 classification, and triggering of an audio output. We present Figure 3 as an operational demonstration of closed-loop performance. All field demonstrations were conducted on private property using standard non-invasive monitoring practices; no animals were handled, baited, or physically contacted.

#### 4.3.2. Illustrative Multi-Species Operation (Ringtail Case Study)

To demonstrate that the same sensing-to-action pipeline can be adapted beyond pumas, we retrained the Stage 2 classifier to identify ringtails using 653 ringtail images. We deployed the system for monitoring ringtails at a residential water feature. Over approximately one month, the system recorded more than 30 ringtail visits and produced 311 usable images. The ringtail-trained Stage 2 binary classifier correctly identified 258 images (approximately 83% image-level accuracy) in this deployment. As expected, accuracy was lower than for pumas since ringtails are fast, small, and the Stage 1 object detector often fails to detect them. When ringtail detections occurred, an audio output was optionally triggered as a demonstration of actuation capability and rapid verification. In some instances the animal left the scene shortly after audio playback; these observations are anecdotal and are included to illustrate closed-loop operation in a different species context. The corresponding video is provided at https://vimeo.com/1120196742 (accessed on 17 February 2026).

### 4.4. End-to-End Latency

We measured end-to-end latency as the elapsed time from camera trigger (image timestamp at upload) to issuance of an action command by the edge device (e.g., notification event and/or initiation of audio playback). This metric captures the practical responsiveness of the system for near-real-time monitoring workflows.

In the current implementation, end-to-end latency is approximately 4 s. Note motion triggering is performed by the camera’s built-in PIR sensor. This value was averaged over 100 triggered events collected during field operation across both networking configurations. This total is dominated by image transfer plus Stage 1 detection/cropping (approximately 3 s) followed by Stage 2 classification (approximately 1 s); other overheads are negligible. In an earlier field demonstration recorded on 19 May 2025 (Figure 3), the end-to-end latency was approximately 8 s; subsequent software optimization (model caching and runtime initialization changes) reduced latency to the current value.

## 5. Discussion

### 5.1. Interpreting Results of the Ablation Study

Key results of the ablation study described in Section 3.2 are summarized in Table 4, which reports both operational FP/FN counts and standard classification metrics (precision, recall, F1, accuracy, balanced accuracy) for each ablation variant.

The Stage 1-only baseline reveals that off-the-shelf detectors are unreliable for species-specific decisions: using the cat proxy produces few false alarms (FP = 7) but misses many true pumas (FN = 319), yielding high precision (0.958) but very low recall (0.334) and a low F1 (0.495), while accuracy (0.773) is dominated by correct no-puma decisions (balanced accuracy = 0.663). This misclassification is not solely a proxy-label artifact; qualitative review shows true pumas frequently assigned to adjacent or unrelated categories (e.g., dog, sheep, horse), and similar label instability occurs even for detector-supported species (e.g., bears) despite correct localization. We attribute this behavior to the detector’s coarse 1000-class taxonomy and training bias toward clear daytime imagery, motivating Stage 1 as a trigger/localizer with species identification deferred to a downstream classifier. From an operational standpoint, Stage 1 is also inexpensive to run on-device (average latency ≈ 0.85 s per image on our Raspberry Pi), but its role must be as a ROI detector rather than species-level classification.

The Stage 2-only baseline (full-image classifier) exhibits the opposite failure mode: it substantially increases false alarms (FP = 171) because motion-triggered field imagery often contains complex backgrounds (vegetation, shadows, precipitation, insects) and, at night, monochrome infrared artifacts and motion blur. This yields high recall (0.931) but lower precision (0.723), so F1 = 0.814; BalAcc = 0.876 remains strong because sensitivity is high, but the reduced specificity implied by 171 FP can be operationally problematic for high-volume deployments. In these conditions, the classifier can confuse background texture or illumination patterns for puma-like features. In addition, without localization the target may occupy only a small fraction of the image or appear as a partial-body capture, which contributes to missed positive events (FN = 33). Together, these effects make full-image classification less reliable for unattended, high-volume triggering in the field. Stage 2-only inference is fast on-device (average latency ≈ 0.55 s per image on our Raspberry Pi), but the absence of localization makes this speed insufficient to offset the operational cost of the elevated false-positive rate.

The “animal-only filter” variant demonstrates why using detector semantic labels as a pre-filter is risky. Although filtering to “animal” labels reduces nuisance ROIs, it also discards many true pumas before Stage 2 is ever applied, increasing missed events.

Finally, the proposed permissive two-stage workflow yields the best overall balance (FP = 8, FN = 12). It is the only variant that simultaneously achieves high precision (0.983) and high recall (0.975), producing the best F1 (0.979) along with accuracy = 0.986 and BalAcc = 0.983. This division of labor reduces false alarms by removing empty or background-dominated images from the classifier’s input distribution, and it reduces missed pumas by concentrating the classifier on localized animal evidence rather than weak, spatially diluted cues in the full image. In operational terms, FP and FN translate directly into workload and risk: the proposed pipeline keeps the “false alert” burden low (8 FP out of 955 no-puma, i.e., high specificity) while preserving sensitivity (only 12 FN out of 479 puma), which is why both accuracy and balanced accuracy are high. The remaining misses are dominated by cases where Stage 1 fails to localize the animal (e.g., low contrast with foliage in nighttime infrared imagery), indicating that localization quality is the primary limiting factor once ROI-based confirmation is employed. In terms of runtime, both two-stage variants (animal-only filter and the proposed permissive Stage 1) have similar end-to-end latency on our Raspberry Pi (average ≈1.5 s per image), which is approximately the Stage 1 cost plus the Stage 2 cost, plus a small handoff/overhead between stages; this is consistent with the Stage 1-only and Stage 2-only latencies reported in Table 2 and Table 3.

### 5.2. Visual Analysis of Illustrative Examples

**Figure 4:**
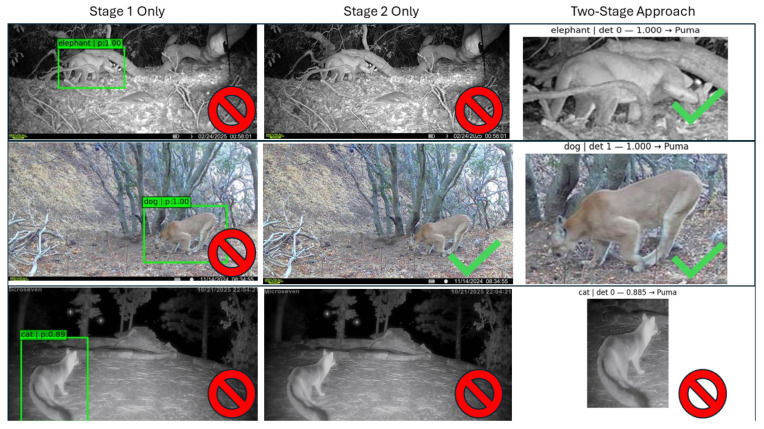
Illustrative examples of the ablation study comparing Stage 1-only, Stage 2-only, and the proposed two-stage pipeline. Columns report the stages and rows include two puma cases (nighttime partially occluded; daytime) and one non-target fox example that produces a puma-like false positive across methods. The first row demonstrates why the two-stage pipeline is necessary for challenging nighttime conditions, whereas the daytime image does not require the first “detect” step. The third row illustrates a rare false positive case across all 3 stages. Red barred circle is failed classification and green check is successful classification.

To complement the quantitative ablation results in Table 4, Figure 4 provides representative examples illustrating the strengths and failure modes of the three configurations (Stage 1 only, Stage 2 only, and the proposed two-stage cascade).

In Figure 4 (row 1), the target is partially obscured against a nighttime monochrome background; Stage 1 localizes the animal but assigns an incorrect label (e.g., “elephant”), consistent with detector label instability under IR illumination, blur, and occlusion. Similar confusion occurs even for native YOLO species (e.g., bears), where localization is often correct but the assigned class drifts to other animals or even non-animal categories, suggesting label confusion is the dominant failure mode rather than the lack of a puma class. Accordingly, Stage 1 is best used as a trigger/localizer, and Stage 2 benefits from cropping: full-image inference can predict no-puma when the animal is small and background-dominated, while the Stage 1 crop enables a confident puma prediction by reducing background confounds.

In the second row, a daytime color image captures a puma in open view with a relatively simple background. Stage 2 (full-image classifier) correctly identifies the target in this easier setting. Stage 1 again localizes the animal but assigns a non-target detector label (e.g., “dog”), reinforcing that Stage 1 should be treated primarily as a localization and candidate-generation stage rather than a species decision rule. The two-stage pipeline correctly classifies the ROI with high confidence, matching the full-image classifier on this straightforward example while retaining the robustness benefits observed in Table 4.

The third row illustrates a challenging non-target false positive: a fox captured at an angle and posture that produces puma-like contours (notably head/shoulder silhouette and tail/torso proportions). All three approaches are misled in this example: Stage 1 assigns a proxy label (e.g., “cat”), Stage 2 applied to the full image predicts puma, and the two-stage pipeline assigns a high puma probability despite operating on the localized ROI. This case demonstrates that false positives are not limited to empty images or nuisance motion; visually similar non-target species and rare poses can also drive errors. The impact of this misidentification is likely small, since, often, the goal is to eliminate a high percentage of false positives, and these types of false positives appear to be rare. In Section 5.3, Grad-CAM visualizations help interpret why the classifier attends to plausible morphological cues in such examples, even when the final decision is incorrect.

### 5.3. Explainability and Error Analysis

**Figure 5:**
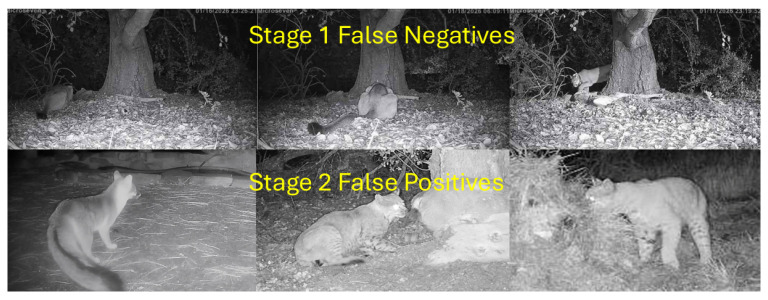
Representative false negatives and false positives observed in field imagery. The top row shows occasional Stage 1 localization failures where the puma the blends into the forest floor or trees due to poor illumination and low contrast leading to missed detections during Stage 1 by YOLO. The bottom row shows foxes and bobcats in poses that exhibit the long tail or other puma like features that fool the EfficientNet binary classifier into false positives. False negatives are typically a result of failure in Stage 1 and can be mitigated by increasing detector sensitivity.

Figure 5 summarizes the dominant failure modes observed in offline evaluation and field operation. Most false negatives arise in Stage 1, where the YOLO detector fails to produce a usable detection for cropping—typically under poor illumination (nighttime IR with low contrast or blur) or when the puma’s appearance blends into the forest floor, logs, or tree textures. In these cases, Stage 2 is never invoked because no ROI is generated. In contrast, the relatively rare false positives are primarily Stage 2 errors: the EfficientNet classifier can be fooled by non-target animals such as foxes and bobcats when viewed at certain angles that resemble puma silhouettes. These false positives are uncommon in practice and have limited operational impact compared to missed detections. Importantly, increasing Stage 1 detector sensitivity improves recall and mitigates many Stage 1 false negatives with little penalty, because additional ROIs passed to Stage 2 are usually rejected by the puma-confirmation classifier. Furthermore, targeted augmentation of the Stage 2 training set with these edge-case poses is a straightforward way to reduce the remaining false positives. This behavior is consistent with field deployments, where some nights produce over 1000 triggered images yet Stage 2 false positives remain infrequent (see Section 4.3).

**Figure 6:**
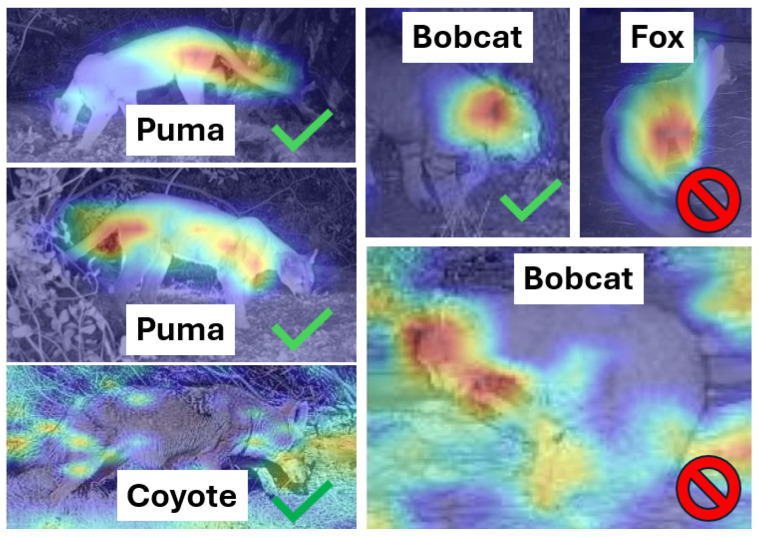
Grad-CAM visualizations for the Stage 2 binary classifier (puma vs. no-puma) on cropped ROI. The panel includes two pumas, one coyote, two bobcats, and one fox. Heatmap intensity indicates feature importance for the classifier decision: red regions contribute most to the predicted class, while low-intensity/no-color regions contribute little. A green check mark indicates a correct prediction; a red barred circle indicates an incorrect prediction. Across puma examples, high-importance regions commonly include the tail, head, torso/shoulder contour, and paws. Misclassifications for visually similar species (e.g., bobcat and fox) often correspond to poses where these cues resemble the puma profile.

We analyzed both model-level and system-level behavior to understand where errors occur in practical deployments. Explainability of the classifier’s decisions serves two purposes. It builds trust in the system in addition to identifying actionable strategies to improve performance. To better understand which image regions drive the Stage 2 binary classifier decisions (puma vs. no-puma), we generated Gradient-weighted Class Activation Mapping (Grad-CAM) visualizations [29]. Grad-CAM uses gradients of the target class score with respect to the final convolutional feature maps to produce a coarse heatmap that highlights regions most influential to the prediction. Figure 6 shows representative heatmaps for multiple species and provides a useful sanity check on what the classifier is using after cropping.

Across many correctly classified puma images (two shown for illustration), the classifier consistently places high importance on anatomically informative cues such as the long tail, head/ear region, torso or shoulder contour, and paws. The heatmaps also help interpret common failure cases. False positives can be explained by overlapping silhouettes and poses (e.g., foxes or coyotes in side-profile with long tails and puma-like silhouettes, and occasional bobcat examples when tail cues are absent or the viewing angle emphasizes head/torso features). These qualitative patterns align with the quantitative ablation results.

### 5.4. Operational Robustness

The device user interface exposes the detector confidence threshold (Stage 1 sensitivity), allowing users to trade off missed detections versus additional candidate ROIs. Increasing sensitivity can reduce missed pumas by accepting weaker detections in low-contrast nighttime imagery; in our experience, this does not substantially increase false alarms because Stage 2 provides strong binary puma confirmation on the cropped regions. This supports a practical deployment strategy: keep Stage 1 permissive to preserve recall, and rely on Stage 2 to maintain specificity.

The deployment software was designed for unattended operation. Corrupted or partially uploaded image files are detected and skipped (logged and ignored) rather than causing pipeline failure. In field operation, the system resumed automatically after brief power or connectivity interruptions once service was restored. We also tested multi-camera operation with up to three cameras connected concurrently; images are processed sequentially via a queue, which keeps peak resource use bounded and is expected to introduce limited backlog in typical wildlife monitoring scenarios where target events are sparse in time.

### 5.5. Limitations and Future Work

Despite these promising results, a few limitations warrant consideration. First, our held-out evaluation provides evidence of generalization for pumas across a meaningful domain shift: training/validation in New Mexico and an independent test in California with different backgrounds and camera hardware. Our training data also span 11 camera brands, introducing substantial variation in infrared appearance and image statistics across devices. Moreover, because Stage 2 operates on cropped animal detections, the classifier is encouraged to learn animal appearance cues rather than site-specific vegetation or scenery. Consistent with pumas having similar visual features across regions, we observed strong performance on the California test set. However, performance may still vary under more extreme shifts in habitat, infrared characteristics, or camera configurations, and a key practical limitation is that many training examples contain clearly visible animals under relatively favorable illumination. Improving robustness to difficult field conditions such as low contrast, partial views, motion blur, or challenging lighting is an important direction for future work.

Second, our multi-species evidence is currently limited to an illustrative ringtail case study, which is intended to demonstrate rapid re-targeting of the same pipeline by retraining only Stage 2 with modest labeled data, rather than to claim universal multi-species performance. A broader, protocol-driven study spanning multiple regions and additional target species with standardized train/test splits across sites and camera models is beyond the scope of this study and is being considered under future work.

Our field validation involved a limited number of puma encounters and a stream that is strongly class-imbalanced, as is typical for camera-trap deployments where non-target triggers dominate. We attempt to compensate for this limitation by reporting metrics that are informative under imbalance (e.g., balanced accuracy and operational false-positive/false-negative rates) rather than relying on accuracy alone.

On the hardware side, although the Raspberry Pi implementation performed reliably in our tests, power interruptions, severe weather, or hardware failures could challenge long-term autonomous deployment. A current field deployment by collaborators from UC Davis is using the same on-device pipeline and is detecting pumas at performance levels consistent with the accuracy reported here, providing an initial external validation under real operating conditions. As a next step, we will couple detection with adaptive responses and evaluate different actions (e.g., deterrent sounds) and their effectiveness in reducing predation events. If there is a need to reduce end-to-end latency in the field while testing deterrence efficacy, we will focus on the largest driver which is the FTP transfer time, rather than the models used for inference. Timing is largely driven by the specific camera hardware and other FTP-enabled camera models may achieve lower transfer latency.

## 6. Conclusions

We presented a deployable, offline vision sensor for near-real-time environmental monitoring that runs on low-cost edge hardware and is designed for challenging field imagery. The core contribution is a practical two-stage pipeline that separates broad localization and empty-image suppression (Stage 1) from target confirmation on ROIs (Stage 2), improving reliability in motion-triggered deployments where most images are empty or non-target. Quantitatively, our strongest evidence of generalization is that puma performance is measured on an independent, geographically disjoint California test set relative to New Mexico training/validation data, indicating robustness to background and camera domain shift.

On a labeled puma vs. no-puma dataset (479 puma, 955 no-puma; N=1434), the proposed two-stage configuration achieved high event-level performance and outperformed single-stage alternatives by reducing false positives caused by complex backgrounds and nuisance triggers. Grad-CAM visualizations further support that, once properly localized, the classifier attends to anatomically meaningful cues for the target decision.

In field deployments operating since May 2025 across two networking modes (site WiFi and standalone hotspot), the system achieves approximately 4 s end-to-end latency from trigger to action command, enabling time-sensitive monitoring use cases in which stakeholders can be notified and events verified while the animal is still present. Field examples demonstrate integrated closed-loop operation with optional actuation outputs.

The system is intended to be reusable across species and sites via retraining of the Stage 2 binary classifier and configurable deployment settings. This was demonstrated by successfully training on a limited number of ringtail images. Ongoing and future work will focus on improving nighttime localization in low-contrast conditions, reducing power demand through camera configurations that avoid continuous capture while retaining event-driven upload, and conducting controlled, protocol-driven evaluations of targeted intervention policies (e.g., audio/light) to quantify behavioral outcomes. More broadly, these observations motivate data-efficient improvement strategies, including model-in-the-loop inspection and edge-enabled feedback loops in which uncertain events are flagged for human review and incorporated into periodic retraining as the system encounters new environments and species.

## Figures and Tables

**Table 1:** Representative power budget for the vision sensor system. Values are typical/observed in our deployment configuration; actual power draw depends on camera model, WiFi conditions, and speaker duty cycle.

Component	Qty.	Power (W)	Notes
Raspberry Pi 5 (edge inference + hotspot + FTP + UI)	1	≈4	Includes local WiFi hotspot and services.
FTP-enabled WiFi PIR security camera	1	≈4	Higher draw due to continuous video and (at night) IR illumination.
USB speaker (optional)	1	<1	Typical draw; depends on volume and playback duty cycle.
**Total (single-camera configuration)**		**≈8**	Observed in our single-camera setup.

**Table 2:** Stage 1-only (YOLO label proxy) comparison between our current YOLOv8 detector [30] and YOLO26 [42]. Metrics are computed on the labeled dataset (479 puma, 955 no-puma; N=1434). Both models perform similarly. The YOLOv8 row is from Equation (4).

Variant	FP	FN	Precision	Recall	F1	Accuracy	BalAcc	Latency (s)
Stage 1 only (YOLOv8 label proxy)	7	319	0.958	0.334	0.495	0.773	0.663	0.85
Stage 1 only (YOLO26 label proxy)	8	316	0.953	0.340	0.502	0.774	0.666	0.75

**Table 3:** Stage 2-only (full-image binary classifier) comparison between EfficientNet [31] and ConvNeXt-Tiny [43]. Metrics are computed on the labeled dataset (479 puma, 955 no-puma; N=1434). Both models perform similarly. The EfficientNet row is from Equation (5).

Variant	FP	FN	Precision	Recall	F1	Accuracy	BalAcc	Latency (s)
Stage 2 only (EfficientNet full image)	171	33	0.723	0.931	0.814	0.858	0.876	≈0.55
Stage 2 only (ConvNeXt-Tiny full image)	165	35	0.729	0.927	0.816	0.861	0.877	≈1.0

**Table 4:** Summary classification metrics for the ablation variants on the labeled dataset (479 puma, 955 no-puma; N=1434). We report standard metrics (precision, recall, F1, accuracy, balanced accuracy) in addition to operationally meaningful FP/FN counts. The proposed workflow demonstrates the best performance across standard metrics.

Variant	FP	FN	Precision	Recall	F1	Accuracy	BalAcc	Latency (s)
Stage 1 only (YOLO label proxy)	7	319	0.958	0.334	0.495	0.773	0.663	0.85
Stage 2 only (full image)	171	33	0.723	0.931	0.814	0.858	0.876	0.55
Two-stage (animal-only filter)	7	89	0.982	0.814	0.890	0.933	0.903	1.5
Two-stage (proposed; permissive Stage 1)	8	12	0.983	0.975	0.979	0.986	0.983	1.5

## Data Availability

The code, training notebooks, and pretrained model weights used in this study are publicly available in the project repository [44]. The labeled image dataset and associated annotations used for the offline experiments are provided through the same repository. Field demonstration media are provided via the links cited in the manuscript. A preconfigured Raspberry Pi microSD image and site-specific configuration files are available upon request.

## Abbreviations

The following abbreviations are used in this manuscript:

AI	Artificial Intelligence
CNN	Convolutional Neural Network
CPU	Central Processing Unit
FTP	File Transfer Protocol
Grad-CAM	Gradient-weighted Class Activation Mapping
IoU	Intersection over Union
IR	Infrared
ML	Machine Learning
NMS	Non-Maximum Suppression
PIR	Passive Infrared (motion sensor)
ROI	Region of Interest
TPU	Tensor Processing Unit
UI	User Interface

## References

[1] Schneider S., Greenberg S., Taylor G.W., Kremer S.C. (2020). Three critical factors affecting automated image species recognition performance for camera traps. Ecol. Evol..

[2] Murphy S.M., Wilckens D.T., Augustine B.C., Peyton M.A., Harper G.C. (2019). Improving estimation of puma (Puma concolor) population density: Clustered camera-trapping, telemetry data, and generalized spatial mark-resight models. Sci. Rep..

[3] Beery S. (2023). The MegaDetector: Large-Scale Deployment of Computer Vision for Conservation and Biodiversity Monitoring.

[4] Ahumada J.A., Fegraus E., Birch T., Flores N., Kays R., O’Brien T.G., Palmer J., Schuttler S., Zhao J.Y., Jetz W. (2020). Wildlife insights: A platform to maximize the potential of camera trap and other passive sensor wildlife data for the planet. Environ. Conserv..

[5] Willi M., Pitman R.T., Cardoso A.W., Locke C., Swanson A., Boyer A., Veldthuis M., Fortson L. (2019). Identifying animal species in camera trap images using deep learning and citizen science. Methods Ecol. Evol..

[6] Tabak M.A., Norouzzadeh M.S., Wolfson D.W., Newton E.J., Boughton R.K., Ivan J.S., Odell E.A., Newkirk E.S., Conrey R.Y., Stenglein J. (2020). Improving the accessibility and transferability of machine learning algorithms for identification of animals in camera trap images: MLWIC2. Ecol. Evol..

[7] Woodroffe R. (2000). Predators and people: Using human densities to interpret declines of large carnivores. Anim. Conserv..

[8] Abrahms B. (2021). Human-wildlife conflict under climate change. Science.

[9] Abrahms B., Carter N.H., Clark-Wolf T., Gaynor K.M., Johansson E., McInturff A., Nisi A.C., Rafiq K., West L. (2023). Climate change as a global amplifier of human–wildlife conflict. Nat. Clim. Change.

[10] Davison A.M., de Koning K., Taubert F., Schakel J. (2025). Automated near real-time monitoring in ecology: Status quo and ways forward. Ecol. Inform..

[11] Whytock R.C., Suijten T., van Deursen T., Świeżewski J., Mermiaghe H., Madamba N., Mouckoumou N., Zwerts J.A., Pambo A.F.K., Bahaa-el din L. (2023). Real-time alerts from AI-enabled camera traps using the Iridium satellite network: A case-study in Gabon, Central Africa. Methods Ecol. Evol..

[12] Yang D., Meng D., Li H., Li M., Jiang H., Tan K., Huang Z., Li N., Wu R., Li X. (2024). A systematic study on transfer learning: Automatically identifying empty camera trap images using deep convolutional neural networks. Ecol. Inform..

[13] Cunha F., dos Santos E.M., Barreto R., Colonna J.G. (2021). Filtering empty camera trap images in embedded systems. 2021 IEEE/CVF Conference on Computer Vision and Pattern Recognition Workshops (CVPRW).

[14] Norouzzadeh M.S., Morris D., Beery S., Joshi N., Jojic N., Clune J. (2021). A deep active learning system for species identification and counting in camera trap images. Methods Ecol. Evol..

[15] Dietrich J., Hollstein A. (2025). Performance and Reproducibility of Large Language Models in Named Entity Recognition: Considerations for the Use in Controlled Environments. Drug Saf..

[16] Huang J., Yang D.M., Rong R., Nezafati K., Treager C., Chi Z., Wang S., Cheng X., Guo Y., Klesse L.J. (2024). A Critical Assessment of Using ChatGPT for Extracting Structured Data from Clinical Notes. npj Digit. Med..

[17] Thomas L.D.W., Romasanta A.K.G., Pujol Priego L. (2026). Jagged Competencies: Measuring the Reliability of Generative AI in Academic Research. J. Bus. Res..

[18] Venugopal S., Gazzetti M., Gkoufas Y., Katrinis K. (2018). Shadow Puppets: Cloud-level Accurate AI Inference at the Speed and Economy of Edge. USENIX Workshop on Hot Topics in Edge Computing (HotEdge).

[19] Redmon J., Divvala S., Girshick R., Farhadi A. (2016). You Only Look Once: Unified, Real-Time Object Detection. 2016 IEEE Conference on Computer Vision and Pattern Recognition (CVPR).

[20] Bochkovskiy A., Wang C.Y., Liao H.Y.M. (2020). YOLOv4: Optimal Speed and Accuracy of Object Detection. arXiv.

[21] Liu W., Anguelov D., Erhan D., Szegedy C., Reed S., Fu C.Y., Berg A.C. (2016). Ssd: Single shot multibox detector. Proceedings of the European Conference on Computer Vision.

[22] Tan M., Pang R., Le Q.V. (2020). Efficientdet: Scalable and efficient object detection. 2020 IEEE/CVF Conference on Computer Vision and Pattern Recognition (CVPR).

[23] Kovachki N., Li Z., Liu B., Azizzadenesheli K., Bhattacharya K., Stuart A., Anandkumar A. (2023). Neural Operator: Learning Maps Between Function Spaces with Applications to PDEs. J. Mach. Learn. Res..

[24] Viswanathan A., Yang X., Tartakovsky D.M. (2026). Fourier Neural Operator Surrogate of Lithium-Ion Battery Models. J. Mach. Learn. Model. Comput..

[25] Donahue J., Anne Hendricks L., Guadarrama S., Rohrbach M., Venugopalan S., Saenko K., Darrell T. (2017). Long-term recurrent convolutional networks for visual recognition and description. IEEE Trans. Pattern Anal. Mach. Intell..

[26] Cho K., van Merriënboer B., Gulcehre C., Bahdanau D., Bougares F., Schwenk H., Bengio Y., Moschitti A., Pang B., Daelemans W. (2014). Learning Phrase Representations using RNN Encoder–Decoder for Statistical Machine Translation. Proceedings of the 2014 Conference on Empirical Methods in Natural Language Processing (EMNLP).

[27] Nazir M., Kaleem S. (2024). Object classification and visualization with edge artificial intelligence for a customized camera trap platform. Ecol. Inform..

[28] Velasco-Montero D., Fernández-Berni J., Carmona-Galán R., Sanglas A., Palomares F. (2024). Reliable and efficient integration of AI into camera traps for smart wildlife monitoring based on continual learning. Ecol. Inform..

[29] Selvaraju R.R., Cogswell M., Das A., Vedantam R., Parikh D., Batra D. (2017). Grad-CAM: Visual Explanations from Deep Networks via Gradient-based Localization. 2017 IEEE International Conference on Computer Vision (ICCV).

[30] Jocher G., Chaurasia A., Qiu J. Ultralytics YOLOv8. GitHub Repository, 2023. Version 8.0.0. https://github.com/ultralytics/ultralytics.

[31] Tan M., Le Q.V. EfficientNet: Rethinking Model Scaling for Convolutional Neural Networks. Proceedings of the 36th International Conference on Machine Learning (ICML).

[32] Deng J., Dong W., Socher R., Li L.J., Li K., Fei-Fei L. (2009). ImageNet: A Large-Scale Hierarchical Image Database. 2009 IEEE Conference on Computer Vision and Pattern Recognition.

[33] Mohammed S.Y. (2025). Architecture review: Two-stage and one-stage object detection. Frankl. Open.

[34] Flutter Is an Open Source Framework for Building Beautiful, Natively Compiled, Multi-Platform Applications from a Single Codebase. https://flutter.dev/.

[35] Smith M.E., Linnell J.D., Odden J., Swenson J.E. (2000). Review of methods to reduce livestock depredation II. Aversive conditioning, deterrents and repellents. Acta Agric. Scand. Sect. A-Anim. Sci..

[36] Zarco-González M., Monroy-Vilchis O. (2014). Effectiveness of low-cost deterrents in decreasing livestock predation by felids: A case in C entral M exico. Anim. Conserv..

[37] Ripple W.J., Estes J.A., Beschta R.L., Wilmers C.C., Ritchie E.G., Hebblewhite M., Berger J., Elmhagen B., Letnic M., Nelson M.P. (2014). Status and ecological effects of the world’s largest carnivores. Science.

[38] Barry J.M., Elbroch L.M., Aiello-Lammens M.E., Sarno R.J., Seelye L., Kusler A., Quigley H.B., Grigione M.M. (2019). Pumas as ecosystem engineers: Ungulate carcasses support beetle assemblages in the Greater Yellowstone Ecosystem. Oecologia.

[39] Elbroch M. (2020). The Cougar Conundrum: Sharing the World with a Successful Predator.

[40] Elbroch L.M., Treves A. (2023). Perspective: Why might removing carnivores maintain or increase risks for domestic animals?. Biol. Conserv..

[41] Cain J.W., Kay J.H., Liley S.G., Gedir J.V. (2024). Mule deer (*Odocoileus hemionus*) Resour. Sel. Trade-Offs Forage Predation Risk. Front. Ecol. Evol..

[42] Sapkota R., Karkee M. (2025). Ultralytics YOLO evolution: An overview of YOLO26, YOLO11, YOLOv8 and YOLOv5 object detectors for computer vision and pattern recognition. arXiv.

[43] Liu Z., Mao H., Wu C.Y., Feichtenhofer C., Darrell T., Xie S. A convnet for the 2020s. Proceedings of the 2022 IEEE/CVF Conference on Computer Vision and Pattern Recognition (CVPR).

[44] Pumaguard v21. https://github.com/PEEC-Nature-Youth-Group/pumaguard/releases/tag/v21.

